# Supplementary material for: The Systems Biology Research Tool: evolvable open-source software
Source: BMC Syst Biol. 2008 Jun 29;2:55. doi: 10.1186/1752-0509-2-55 (PMC2446383; doi:10.1186/1752-0509-2-55)
Supplement: Additional file 1 — SBRT Archive. An archive of the current version of the Systems Biology Research Tool. [file 1752-0509-2-55-S1.zip › sbrt-1.4.0/doc/developers_guide/api/sbrt/shell/text/PercentFormatVersion1.html]

PercentFormatVersion1


|  |  |  |
| --- | --- | --- |
| **Overview** \| **Package** \| **Class** \| **Use** \| **Tree** \| **Deprecated** \| **Index** \| **Help** | |  |
| **PREV CLASS**   **NEXT CLASS** | **FRAMES**    **NO FRAMES**     **All Classes** |  |
| SUMMARY: NESTED \| FIELD \| CONSTR \| METHOD | DETAIL: FIELD \| CONSTR \| METHOD |  |


---


## sbrt.shell.text Class PercentFormatVersion1

```
java.lang.Object
  sbrt.shell.text.PercentFormatVersion1
```

**All Implemented Interfaces:**: Format, Formatter<java.lang.Double>, Parser<java.lang.Double>, PercentFormat, SimpleFormat<java.lang.Double>

---

``` public class PercentFormatVersion1 extends java.lang.Object implements PercentFormat ```

This class is a concrete implementation of
`PercentFormat`.

**Author:**
:   This class was written and documented by
    Jeremiah Wright while in the Wagner lab.

---

**Constructor Summary**

`PercentFormatVersion1()`
  
          Constructs a new percent format.


| **Method Summary** | |
| --- | --- |
| `java.lang.String` | `format(double percentage)`             Returns a formatted string representation of the provided percentage. |
| `java.lang.String` | `format(java.lang.Double percentage)`             Returns a formatted string representation of the provided percentage. |
| `DoubleFormatV1` | `getDoubleFormat()`             Returns the `DoubleFormat` used to parse and format the numerical percentage values. |
| `java.lang.Double` | `parse(java.lang.String percentage)`             Parses the provided string and returns its corresponding percentage. |
| `double` | `parsePrimitive(java.lang.String percentage)`             Parses the provided string and returns its corresponding percentage. |

**Methods inherited from class java.lang.Object**

`clone, equals, finalize, getClass, hashCode, notify, notifyAll, toString, wait, wait, wait`

**Constructor Detail**

### PercentFormatVersion1

```
public PercentFormatVersion1()
```

:   Constructs a new percent format.


**Method Detail**

### getDoubleFormat

```
public DoubleFormatV1 getDoubleFormat()
```

:   Returns the `DoubleFormat` used to
    parse and format the numerical percentage values.

    :   **Returns:**: the `DoubleFormat` used to parse and format the numerical percentage values.

---


### format

```
public java.lang.String format(double percentage)
```

:   Returns a formatted string representation of the
    provided percentage. The percentage is formatted
    as a double precision number, truncated after
    the second decimal place, and followed by the
    `%` sign.

    :   **Specified by:**: `format` in interface `PercentFormat`
    :   **Parameters:**: `percentage` - the percentage to be formatted. **Returns:**: a formatted string representation of the provided percentage.

---


### format

```
public java.lang.String format(java.lang.Double percentage)
```

:   Returns a formatted string representation of the
    provided percentage. The percentage is formatted
    as a double precision number, truncated after
    the second decimal place, and followed by the
    `%` sign.

    :   **Specified by:**: `format` in interface `Formatter<java.lang.Double>` **Specified by:**: `format` in interface `PercentFormat`
    :   **Parameters:**: `percentage` - the percentage to be formatted. **Returns:**: a formatted string representation of the provided percentage.

---


### parse

```
public java.lang.Double parse(java.lang.String percentage)
```

:   Parses the provided string and returns its
    corresponding percentage.

    :   **Specified by:**: `parse` in interface `Parser<java.lang.Double>` **Specified by:**: `parse` in interface `PercentFormat`
    :   **Parameters:**: `percentage` - the string to be parsed. **Returns:**: the double precision number corresponding to the provided string.

---


### parsePrimitive

```
public double parsePrimitive(java.lang.String percentage)
```

:   Parses the provided string and returns its
    corresponding percentage.

    :   **Specified by:**: `parsePrimitive` in interface `PercentFormat`
    :   **Parameters:**: `percentage` - the string to be parsed. **Returns:**: the double precision number corresponding to the provided string.


---


|  |  |  |
| --- | --- | --- |
| **Overview** \| **Package** \| **Class** \| **Use** \| **Tree** \| **Deprecated** \| **Index** \| **Help** | |  |
| **PREV CLASS**   **NEXT CLASS** | **FRAMES**    **NO FRAMES**     **All Classes** |  |
| SUMMARY: NESTED \| FIELD \| CONSTR \| METHOD | DETAIL: FIELD \| CONSTR \| METHOD |  |


---
